# Antidiabetic and Other Therapies Used in Subjects with Diabetes and Chronic Kidney Disease in a Hospital-Based Clinic Population in Greece

**DOI:** 10.3390/jcm10102104

**Published:** 2021-05-13

**Authors:** Ilias N. Migdalis, Nikolaos Papanas, Ioannis M. Ioannidis, Alexios E. Sotiropoulos, Athanasios E. Raptis, George D. Dimitriadis, on behalf of the Hellenic Diabetic Nephropathy Study (HDNS)

**Affiliations:** 1 Second Medical Department and Diabetes Centre, NIMTS Hospital, 11521 Athens, Greece; 2 Second Department of Internal Medicine and Diabetes Centre, University Hospital of Alexandroupolis, Democritus University of Thrace, 68132 Alexandroupolis, Greece; papanasnikos@yahoo.gr; 3 First Department of Internal Medicine and Diabetes Centre, General Hospital of Nea Ionia Konstantopoulio-Patision, 14233 Athens, Greece; kefion@otenet.gr; 4 Third Medical Department and Diabetes Centre, Hospital of Nikaia, 18454 Piraeus, Greece; alexiosotiropoulos@gmail.com; 5 Second Department of Internal Medicine, Attikon University Hospital, Medical School, National and Kapodistrian University of Athens, 12462 Athens, Greece; atraptis@med.uoa.gr (A.E.R.); gdimitr@med.uoa.gr (G.D.D.)

**Keywords:** diabetes mellitus, diabetic chronic kidney disease, diabetic nephropathy

## Abstract

(1) Background: Type 2 diabetes mellitus (T2DM) is the main cause of chronic kidney disease (CKD). In Greece, in a population from hospital-based diabetes clinics (*n* = 1759), the overall prevalence of diabetic chronic kidney disease (DCKD) was 45% including mild, moderate, and severe CKD. The aim of this study was to describe and analyze how T2DM patients with mild-to-severe CKD are managed by diabetologists in Greece and assess the achievement rates in glycemic, blood pressure and low-density lipoprotein-cholesterol (LDL-C) control. (2) Methods: This cross-sectional multicenter study took place from June 2015 to March 2016 and collected data from diabetes centers in public hospitals all over Greece. (3) Results: With regard to the anti-diabetes treatment, most participants were on metformin, DPP-4 (Dipeptidyl Peptidase-4 inhibitors) inhibitors and insulin. Angiotensin-converting enzyme inhibitors or angiotensin II receptor blockers were the most prescribed medications for hypertension. For the management of dyslipidemia, most participants were on statins. For patients with DCKD, the levels of HbA1c, blood pressure and LDL-C were 7.2%, 137.7/76.9 mmHg and 95.9 mg/dL, respectively (mean values). (4) Conclusions: The outcomes of this study suggest that management of DCKD can be further improved and should be enhanced. These results may contribute to the whole health care system in Greece. In addition, the better understanding of therapeutic strategies used by diabetologists treating these patients offers educational benefits to primary care physicians, which can result in an overall more successful and efficient management of subjects with T2DM and DCKD.

## 1. Introduction

Worldwide studies show that chronic kidney disease (CKD) is a leading cause of end-stage renal disease (ESRD) in type 2 diabetes mellitus (T2DM), which also increases cardiovascular mortality [1]. We previously reported a 45% overall prevalence of CKD (mild, moderate and severe) in a hospital-based T2DM population [2]. There is still some uncertainty on the true benefits of tight glycemic control on CKD progression, but antidiabetic, antihypertensive and hypolipidemic treatment remains the cornerstone of management [3,4]. Within this context, the choice of suitable therapeutic agents and their use in clinical practice is still a matter of worldwide interest. The present study was designed to examine which antidiabetic, antihypertensive and hypolipidemic agents are used in subjects with diabetes and CKD in Greece, looking at a hospital-based clinic population. The results of this study reflect current choices of antidiabetic, antihypertensive and lipid-lowering agents by specialists aiming: (a) to critically assess their clinical use and see how this could set an example, and (b) to highlight the need for polypharmacy. The findings could be useful for non-specialists and general practitioners since gradual deterioration of kidney function is an important comorbidity to be considered when selecting antidiabetic agents.

## 2. Methods and Data Analysis

The study was conducted from June 2015 to March 2016 and included consecutive adult T2DM subjects regularly attending hospital-based diabetes centers and clinics in Greece [2]. Within a period of 6 months prior to recruitment, laboratory data (urea, creatinine, albuminuria, urine albumin/creatinine ratio) were acquired for all participating patients. The study was approved by the institutional committees and participants gave their informed consent. Glomerular filtration rate (GFR) was calculated according to MDRD (Modification of Diet in Renal Disease) [5]. CKD severity was staged according to KDIGO criteria (Kidney Disease: Improving Global Outcomes) [6]. All subjects were classified according to eGFR (mg/mL/1.73 m^2^): G1 > 90, G2 60–89, G3a 45–59, G3b 30–44, G4 15–29, G5 < 15. They were also classified into the following categories according to the Albumin/creatinine ratio (mg/gr Cr): A1 < 30, A2 30–300, A3 > 300. Categories G1A1 and G2A1 were considered normal. Given the size of our sample and the difficulty to adequately represent the 18 CKD stages, four new categories were coined as follows: normal kidney function, mild CKD, moderate CKD and severe CKD (Table 1). During entry visits, we measured weight, height, and blood pressure (BP). Age, diabetes duration, anti-diabetic and anti-hypertension treatment, current smoking (yes or no) and other lifestyle factors (e.g., alcohol use, exercise), as well as diabetic macro- or microvascular disease were documented according to medical records.

### Statistical Analysis

The statistical package R (version 3.2.5-R Core Team) was used. For numerical variables, data are presented as means and standard deviations (SD). Categorical variables are described as numbers and percentages. Stages for kidney function were regrouped into four and then two categories for modelling purposes: (i) four categories: normal kidney function, mild, moderate, and severe CKD; (ii) two categories: normal kidney function vs. CKD. Univariate analysis was originally performed. Multiple analyses were then employed to associate CKD risk with treatment regimen (antidiabetic, antihypertensive and hypolipidemic therapy). The proportional odds methodology was used to quantify the relationship of CKD with treatment. Significant covariates were selected using backward stepwise selection. Significance was determined at 5% level (two-tailed *p* < 0.05).

## 3. Results

In the entire population (*n* = 1759), the mean age was 68 years, 54.6% were male and mean T2DM duration was 13 years. The overall prevalence of DCKD was 45% distributed to stages from mild to severe with mean CKD duration 4.1 years. Most participants (*n* = 1363, 77%) had hypertension, 1372 (77.9%) had dyslipidemia and 300 (17%) were current smokers. Mean body mass index (BMI) was 30.9 kg/m^2^.

Patients with DCKD compared with patients without DCKD had higher mean age (61.1 vs. 64.9, *p* < 0.01), longer duration of diabetes (15.3 vs. 11.8 years, *p* < 0.01), and were more likely to be men (*p* < 0.01). Moreover, patients with DCKD had higher mean HbA1c and SBP (*p* < 0.01). For patients with DCKD, mean HbA1c, SBP, DBP and LDL-C levels were 7.2%, 137.7 mmHg, 76.9 mmHg and 95.9 mg/dL, respectively (Table 1).

**Figure 1:**
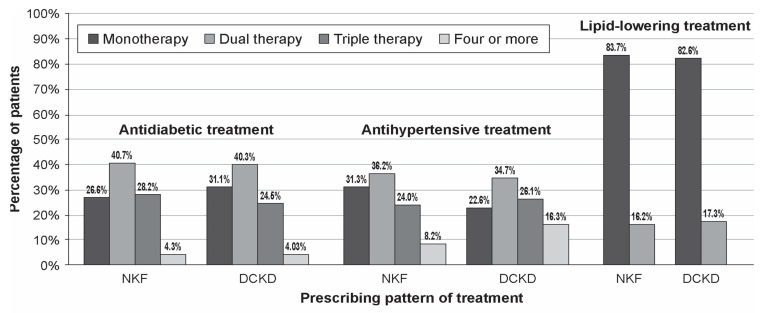
Prescribing pattern of treatment (NKF: normal kidney function, DCKD: diabetic chronic kidney disease).

Regarding the antidiabetic treatment, most participants were on metformin (*n* = 1438, 81.8%), DPP-4 inhibitors (*n* = 716, 40.7%) and insulin (*n* = 716, 40.8%). Metformin was most commonly administered to mild and moderate DCKD, while insulin was mostly administered to severe DCKD. Angiotensin-converting enzyme inhibitor (ACE-I) or an angiotensin II receptor blocker (ARB) were the most commonly prescribed antihypertensive medications, followed by calcium channel blockers (CCBs), B-blockers and diuretics. For the management of dyslipidemia, most participants were treated with statins (*n* = 1690, 96%), followed by ezetimibe (*n* = 181, 10.2%) (Table 2). Most of the participants received more than one antidiabetic or antihypertensive medication (71.3% and 72.5%, respectively) while the majority (83.2%) of the participants received only statins as lipid-lowering treatment (Table 3, Figure 1).

## 4. Discussion

Pharmacological treatment of DCKD is primarily targeted towards lowering HbA1c and blood pressure [7]. Moreover, the prevalence of cardiovascular disease is significantly higher among individuals with DCKD compared to those without. Therefore, treatment with a low-to-moderate-dose statin is suggested for patients with DCKD aged 50 years or older, irrespective of low-density lipoprotein cholesterol level [8,9]. Smoking cessation should also be encouraged [10,11]. In patients with DCKD, a reduction of multiple risk factors is also needed; this may result in a necessary polypharmacy. In Greece, since all agents are reimbursed, the choice of drugs by the specialists is not expected to have influenced the results.

Glycemic targets in the presence of DCKD should be personalized in accordance with the patient characteristics. Glycemic control may delay progression of DCKD, with most guidelines recommending a goal HbA1c around or below 7.0% [8,12,13,14,15,16]. Studies in patients with T2DM, including the Veterans Affairs Diabetes Trial (VADT) [17], the Action in Diabetes and Vascular Disease, Preterax and Diamicron MR Controlled Evaluation (ADVANCE) trial [18] and the Action to Control Cardiovascular Risk in Diabetes (ACCORD) trial [19] have provided convincing evidence that intensive glycemic control reduces albuminuria but with no significant improvement in DCKD progression. However, evidence in these studies shows that intensive glycemic control did not decrease the risk of cardiovascular disease or kidney events (doubling of serum creatinine or ESKD) and all-cause mortality was increased in ACCORD [17,18,19]. Therefore, an HbA1c target <7% is not recommended in patients with an increased risk for hypoglycemia (e.g., those treated with insulin or sulphonylureas or being in DCKD stage 4 or 5) [20].

There are several factors that predispose patients with DCKD to an increased risk of hypoglycemia. Patients with decreased GFR (<60 mL/min/1.73 m^2^) due to DCKD have decreased insulin requirements, as insulin is cleared by the kidneys [21]. In DCKD patients, the peripheral metabolism of insulin is reduced [22]. In patients with DCKD and uremia, anorexia can cause suboptimal nutrition and decreased liver glycogen storage [23]. Moreover, in DCKD patients, reduction in renal mass can lead to reduced renal gluconeogenesis [22]. In a retrospective analysis of 240,000 subjects, the incidence of hypoglycemia was higher in patients with versus without DCKD and the odds of mortality were increased at all levels of hypoglycemia, constituting a significant threat [24].

In our study, mean HbA1c levels for patients with normal kidney function were 7.0%, and 7.2% for patients with mild to severe DCKD. The frequency of inadequate glycemic control is commonly around 50% and even as high as 76% [25,26,27]. Several studies have examined the level of glycemic control in T2DM in Greece: (a) in a retrospective observational study, the proportion of patients achieving the target of HbA1c <7% was 53.9% in 2012 and 56.1% in 2006 [28]. (b) In a retrospective cross-sectional study, HbA1c <7% was achieved by 42% of the patients [29]. (c) In a cross-sectional study conducted in 15 hospital-based outpatient clinics and 38 private practices located across Greece, HbA1c target was achieved by 82.1% of the subjects [30]. (d) A cross-sectional disease registry of current T2DM management, representing the majority of Diabetes Centers or Diabetes Outpatients Clinics, showed that the mean (±SD) HbA1c of the total population was 7.1 ±1.2% [31]. In these trials, the doctors participating were diabetes specialists and the results agree with those in our study. However, the management of patients with T2DM and DCKD in primary care is not widely known. A publication by Grandfils et al. [32] showed that, in their therapeutic decision-making, general practitioners most commonly consider the severity of T2DM, but not that of CKD.

The choice of antidiabetic regimen for patients with T2DM and CKD relies on an interplay of patient characteristics, low risk of hypoglycemia and weight gain, and the availability of therapeutic options [33]. It may be essential to introduce dose adjustments in oral hypoglycemic agents when treating DCKD. Clinicians must be aware that as kidney function worsens, abnormalities in glucose homeostasis develop, affecting secretion, clearance, and tissue sensitivity to insulin. A study by Ruzafa et al. [34] using data from the UK primary care setting reported that in subjects with T2DM and CKD, estimated glomerular filtration rate progressed one stage over 1.7–1.9 years of follow-up. In another study, CKD worsening was observed in approximately 10–17% of T2DM patients over a median follow-up of 2 years [35]. In such cases, accumulation of uremic toxins induces chronic inflammation, excess visceral fat, oxidative stress and metabolic acidosis; these can all affect the insulin signaling pathway and induce insulin resistance by attenuating the ability of insulin to suppress hepatic gluconeogenesis and glucose production [36,37,38,39,40].

In most, cases, metformin is the first-choice antidiabetic meditation. In our study, metformin was administered mostly to low stages of DCKD, as opposed to insulin which was mostly given to more severe DCKD. It has been reported that patients with T2DM and eGFR 45–60 mL/min/1.73 m^2^ experience a lower risk of all-cause mortality with metformin administration [41]. The most common adverse reactions to metformin concern the gastrointestinal system, but the most feared reaction, although rare, is lactic acidosis [42]. However, there is no evidence from prospective comparative or observational studies that metformin increases the risk of lactic acidosis. The latter was reported in a meta-analysis from 347 comparative trials and cohort studies [43]. The US Food and Drug Administration (FDA) guidelines state that metformin is contraindicated in patients with an eGFR <30 mL/min/1.73 m^2^. Metformin dosage may require modification with an eGFR <60 mL/min/1.73 m^2^ [25]. Risk of hypoglycemia with sulphonylureas is increased in patients with DCKD because the reduced GFR prolongs their pharmacodynamic action. Since T2DM patients with CKD often have other comorbidities and may be old and frail, safer treatment alternatives are preferred [44]. A study in 21 individuals with moderate to severe DCKD and six healthy volunteers receiving single or multiple doses of pioglitazone 45 mg revealed no significant accumulation of the drug and its metabolites [45]. Pioglitazone can be used without dose adjustment but it is associated with a risk of weight gain, congestive heart failure and bone fractures [46]. In trials lasting ≥24 weeks, the mean reduction in HbA1c with DPP-4 inhibitors was typically 1.1 to 0.6% [47]. DPP-4 inhibitors are no more efficient in lowering blood glucose concentrations and reducing HbA1c levels than the older molecules and they provide certain advantages such as an insignificant risk of hypoglycemia and a weight-neutral profile. In clinical trials lasting ≥16 weeks in patients with DCKD, GLP-1 receptor agonists reduced the mean HbA1c from baseline by 1.4% to 0.7% [7,48]. The glycemic efficacy of SGLT2 inhibitors decreases with a worsening degree of DCKD. In clinical trials in patients with DCKD up to stage 3, SGLT2 inhibitors have shown moderate glucose-lowering efficacy with a mean reduction in HbA1c ranging from 0.6% to 0.3% [49]. In patients with more advanced DCKD, the use of SGLT2 inhibitors is not recommended due to reduced efficacy. In addition, the use of specific medication classes such as SGLT2 inhibitors in those with severely increased albuminuria should be considered. The Canagliflozin and Renal Events in Diabetes and Established Nephropathy Clinical Evaluation (CREDENCE) trial demonstrated that among 4401 patients with T2DM and CKD stage G2-G3/A3 (baseline eGFR 30 to <90 mL/min/1.73 m^2^ and urine ACR >300–5000 mg/24 h) taking angiotensin-converting enzyme inhibitor (ACE-1) or angiotensin II receptor blocker (ARB) therapy, those randomized to canagliflozin had a 30% lower risk (43.2 vs. 61.2 events per 1000 patient-years) of developing the primary composite renal outcome (doubling of serum creatinine, ESRD or death from a renal or cardiovascular cause) compared with those randomized to placebo [50]. The benefits offered by this class of medications have also been suggested by earlier trials, which could extend to patients with DCKD with lower levels of albuminuria [51,52]. In particular, it seems that that more substantial evidence of nephroprotective effects has been shown in studies with the use of SGLT2 inhibitors and GLP-1 receptor agonists [53,54,55]. According to the recent guidelines of the American Diabetes Association, nephroprotective effects were reported for three SGLT2 inhibitors (empagliflozin, canagliflozin and dapagliflozin) and three GLP-1 receptor agonists (liraglutide, semaglutide and dulaglutide) [16]. Finally, it should be pointed out that the changes in the dosage of insulin should be decided based on the estimation of glycemic fluctuations rather than kidney function [56].

Current evidence highlights the importance of BP reduction in the management of patients with DCKD. The rates of cardiovascular and kidney events increase when BP is elevated, whereas they progressively decrease when BP is kept within normal limits with the appropriate treatment. We should note, however, that there may be a point beyond which further BP reduction may not be helpful or even be harmful despite a reduction in proteinuria. The current KDOQI guidelines recommend a goal of BP <130/80 mmHg, whereas the Eighth Joint National Committee (JNC8) guidelines recommend a goal of BP <140/90 mmHg for most patients with T2DM and diabetic nephropathy, but with individualization [3,57,58,59,60,61]. In our study, most participants (77%) had hypertension. The mean BP for patients with normal kidney function was 130.7/76.9 mmHg and 137.7/76.9 mmHg for patients with mild to severe DCKD. According to a survey conducted in the United States, 35% of patients with hypertension had their BP under control [62]. In the routine care setting in Greece, BP target was achieved by 42.6% of subjects with T2DM [30]. In our study, blockade of the rennin-angiotensin-aldosterone system with either an angiotensin-converting enzyme inhibitor (ACE-1) or an angiotensin II receptor blocker (ARB) were the most frequent antihypertensive agents in all stages of CKD. These agents are recommended for adults with diabetes and a urine albumin-to-creatinine ratio (ACR) of at least 30 mg per 24 h or any adult with a urine ACR of at least 300 mg per 24 h [8,14,63]. In agreement with previous findings, it took two or more BP medications to achieve the goal of BP levels in our patients. The landmark ACCORD trial tested the hypothesis that more intensive BP control (systolic BP <120 mmHg) would be of benefit compared to standard BP therapy (systolic BP <140 mmHg). In this randomized trial with 4.733 patients, an average of 3.5 BP medications were used to achieve the above BP goal in the intensive therapy group compared to 2.5 BP medications used in the standard therapy group [64].

Because dyslipidemia is closely related to the progression of cardiovascular disease, treatment is warranted irrespective of its potential effect on diabetic nephropathy. Since the risk of cardiovascular disease is excessive in subjects with diabetes, recommendations of an aggressive lowering of LDL cholesterol levels, given for patients with established cardiovascular disease, can also be extended to those with diabetes [65]. In our study, most participants (77.9%) had dyslipidemia. The mean LDL-C for patients with normal kidney function was 98.4 mg/dL, and 95.9 mg/dL in patients with mild to severe DCKD. The most frequent agent used were statins (95.5% in DCKD subjects, 95.8% in normal kidney function subjects). Combination therapy for LDL-C lowering with statins and ezetimibe was 11.4% and 10.2%, respectively. In the routine care setting in Greece, LDL-C targets were achieved in 57.0% of the patients [30]. Randomized controlled trials have indicated that statins may preserve renal function. As a result, a greater degree of renal preservation is seen in patients treated with higher doses of statin; these patients also had the biggest reductions in LDL-cholesterol and triglycerides [66]. However, in the Atorvastatin Study for Prevention of Coronary Artery Disease Endpoints in Non-Insulin Dependent Diabetes Mellitus (CARDS) trial, treatment with atorvastatin was not associated with progression or regression of albuminuria [67]. Along with statins, other lipid-lowering therapies, such as fibrates, are also important. It should be noted however, that the combination of statins and fibrate is contraindicated for patients with moderate to severe kidney impairment. An effective combination is statins with ezetimibe. Of course, in all cases, the clinical background of each patient should be considered before starting a lipid-lowering therapy.

In this setting, such therapeutic choices are not only relevant to Greece but also to other countries. It should also be stressed that patients with DCKD should avoid natural products and other over-the-counter supplements since there may be unwanted interactions with prescribed medications or other side effects. However, a collection of data on this issue was not included in our study. A previous publication from our team found a significant correlation of DCKD with retinopathy and peripheral neuropathy [2]. Therefore, in contrast to previous publications [32,33,39,61], a more holistic approach to the management of patients with DCKD is proposed by the present study, including not only the regulation of hyperglycemia but also that of BP and dyslipidemia. According to the recent American Diabetes Association Guidelines, when patients have an eGFR <30 mL/min/1.73 m^2^, they should be referred to a nephrologist [16].

The occurrence and the progression of DCKD can be slowed down markedly, even in advanced stages of kidney disease. The management of CDKD is complex and should be personalized, involving the patients in the treatment process. Physicians should therefore consider the interplay between the psychosocial background, the comorbidities and the pharmacokinetic/pharmacodynamic characteristics of the anti-diabetic drugs when deciding the appropriate therapeutic regimens for their patients to achieve what is desirable and what is practically possible.

The strengths of this study include the nationwide and real-world design and the enrollment of consecutive cases throughout Greece. There may be some selection bias inasmuch as subjects are restricted to diabetes units in hospitals. However, it should be pointed out that the majority of our T2DM subjects are residents of large cities and, due to the recent economic crisis, they are mostly treated in public hospitals rather than private practices [2]. Furthermore, there was a limited use of SGLT2 inhibitors in our patients since, at the time of recruitment (2015–2016), the multicenter studies on the beneficial effects of this class of drugs in DCKD were not yet available.

## 5. Conclusions

The presence of kidney disease adds an extra layer of complexity to the management of T2DM patients compared to those with diabetes alone. Optimal management of DCKD includes treatment of T2DM and hypertension, cardiovascular risk reduction and adjustments to drug dosing. The outcomes of this study suggest that management of DCKD can be further improved and should be enhanced. These results can contribute to the whole health system in Greece. In addition, a better understanding of the therapeutic strategies used by diabetologists treating these patients offers educational benefits to primary care physicians, which can result in an overall more successful and efficient management of T2DM patients with DCKD.

## Figures and Tables

**Table 1:** Patient demographics and disease characteristics. Two-group analysis (normal kidney function vs. DCKD). Values are presented as a percentage (%) of the total number or as a mean ± standard deviation (SD). DCKD: Diabetic Chronic Kidney Disease, BMI: Body Mass Index, CKD: Chronic Kidney Disease, SBP: Systolic Blood Pressure. DBP: Diastolic Blood Pressure, LDL-C: Low Density Lipoprotein-Cholesterol.

Redit-2-Diag Sample, *n*	Normal Kidney Function 965	Mild DCKD 539	Moderate DCKD 135	Severe DCKD 120	Total 1759	Odds	95% CI
Sex (males)	487 (50.5%)	298 (55.3%)	77 (57%)	67 (55.8%)	929 (54.6%)	1.22	1.02–1.46
Age (years) mean (SD)	64.9 (9.4)	66.8 (9.3)	69.8 (8.3)	70.8 (8.9)	68 (9.5)	1.04	1.03–1.05
BMI mean (SD)	30.7 (5.7)	31.1 (5.7)	31.3 (6.0)	30.9 (5.6)	30.9 (5.7)	1.01	1.00–1.03
Current smokers	166 (17.2%)	102 (18.9%)	13 (9.6%)	19 (15.8%)	300 (17%)	1.01	1.00–1.02
Diabetes duration mean, years, (SD)	11.8 (8.2)	13.9 (8.5)	14.9 (8.1)	17.1 (9.9)	13 (8.5)	1.04	1.03–1.05
CKD duration mean, years, (SD)		3.7 (4.1)	4.0 (3.6)	4.7 (3.5)	4.1 (3.6)		
Mean HbA1c, %, (SD)	7.0 (1.1)	7.1 (1.2)	7.2 (1.2)	7.3 (1.3)	7.1 (1.2)	1.14	1.06–1.23
Mean SBP, mmHg, (SD)	130.7 (14.9)	136.1 (16.7)	138.7 (17.9)	138.4 (18.7)	135.9 (17)	1.26	1.19–1.33
Mean DBP, mmHg, (SD)	76.9 (9.2)	77.4 (10.6)	77.4 (10.9)	75.9 (10.4)	76.9 (9.9)	1.01	0.92–1.11
Mean LDL-C, mg/dL, (SD)	98.4 (32.0)	98.8 (32.9)	94.6 (29.6)	94.3 (35.0)	96.5 (32.8)	1.00	1.00–1.00

**Table 2:** Pharmacological treatment. Two-group analysis (normal kidney function vs. DCKD). Abbreviations: ACE-I, angiotensin-converting enzyme inhibitor; ARBs, angiotensin II receptor blockers; CCBs, calcium channel blockers; Aldosterone RA, aldosterone receptor antagonists.

Redit-2-Diag Sample, *n*	Normal Kidney Function 965	Mild DCKD 539	Moderate DCKD 135	Severe DCKD 120	Total 1759	Odds	95% CI
**Antidiabetic treatment**							
Metformin	860 (89.1%)	436 (80.9%)	92 (68.1%)	50 (41.7%)	1438 (81.8%)	0.32	0.25–0.42
DPP-inhibitors	412 (42.7%)	207 (38.4%)	53 (39.3%)	44 (36.7%)	716 (40.7%)	0.85	0.70–1.02
Sulfonylureas	229 (23.7%)	132 (24.5%)	34 (25.2%)	15 (12.5%)	410 (23.3%)	0.93	0.75–1.16
Pioglitazone	65 (6.7%)	21 (3.9%)	5 (3.7%)	3 (2.5%)	94 (5.3%)	0.54	0.35–0.84
SGLT2 inhibitors	25 (2.6%)	15 (2.8%)	3 (2.2%)	1 (0.8%)	44 (2.5%)	0.92	0.51–1.65
Insulin	324 (33.6%)	235 (43.6%)	77 (57%)	81 (67.5%)	717 (40.8%)	1.96	1.63–2.37
GLP-1 analogs	91 (9.4%)	32 (5.9%)	9 (6.7%)	11 (9.2%)	143 (8.1%)	0.68	0.48–0.97
**Antihypertensive Treatment**							
ACE-I	256 (26.5%)	157 (29.1%)	32 (23.7%)	22 (18.3%)	467 (26.5%)	0.99	0.78–1.25
ARBs	583 (60.4%)	321 (59.5%)	74 (54.8%)	66 (55.0%)	1044 (59.35%)	0.90	0.73–1.11
CCBs	322 (33.3%)	233 (43.2%)	72 (53.3%)	73 (60.8%)	700 (39.7%)	1.63	1.30–2.04
B-Blockers	368 (38.1%)	227 (42.1%)	69 (51.1%)	45 (37.5%)	709 (40.9%)	1.21	0.98–1.50
Thiazide diuretics	362 (37.5%)	214 (39.7%)	48 (35.5%)	34 (28.3%)	658 (37.4%)	0.99	0.80–1.23
Aldosterone RA	26 (2.7%)	12 (2.2%)	8 (5.9%)	3 (2.5%)	49 (2.7%)	1.14	0.61–2.13
Loop diuretics	56 (5.8%)	42 (7.7%)	23 (17%)	35 (29.1%)	156 (8.8%)	2.49	1.71–3.64
**Lipid-lowering agents**							
Statins	925 (95.8%)	524 (97.2%)	123 (91.1%)	118 (98.3%)	1690 (96.0%)	1.31	0.74–2.32
Ezetimibe	99 (10.2%)	51 (9.4%)	9 (6.6%)	22 (18.3%)	181 (10.2%)	1.00	0.71–1.41
Fibrates	57 (5.9%)	26 (4.8%)	14 (10.3%)	5 (4.1%)	102 (5.7%)	1.06	0.69–1.65

**Table 3:** Prescribing pattern of antidiabetic and antihypertensive agents in the two-group analysis (normal kidney function vs. DCKD).

Redit-2-Diag Sample, *n*	Normal Kidney Function 965	Mild DCKD 539	Moderate DCKD 135	Severe DCKD 120	Total1 759	Odds	95% CI
**Antidiabetic Treatment**							
Mono therapy	257 (26.6%)	155 (28.7%)	40 (29.6%)	52 (43.3%)	504 (28.6%)	1.58	1.13–2.00
Dual therapy	393 (40.7%)	222 (41.1%)	52 (38.5%)	46 (38.3%)	713 (40.5%)	0.87	0.69–1.09
Triple therapy	273 (28.2%)	142 (26.3%)	38 (28.1%)	15 (12.5%)	468 (26.6%)	0.76	0.59–0.98
Four or more	42 (4.3%)	20 (3.7%)	5 (3.7%)	7 (5.8%)	74 (4.2%)	0.76	0.46–1.27
**Antihypertensive Treatment**							
Mono therapy	303 (31.3%)	134 (24.8%)	22 (16.2%)	24 (20.0%)	483 (27.4%)	1.40	1.10–2.00
Dual therapy	350 (36.2%)	187 (34.6%)	48 (35.5%)	41 (34.1%)	626 (35.5%)	1.31	1.00–1.72
Triple therapy	232 (24.0%)	138 (25.6%)	38 (28.1%)	32 (26.6%)	440 (25.0%)	1.45	1.08–1.94
Four or more	80 (8.2%)	80 (14.8%)	27 (20.0%)	23 (19.1%)	210 (11.9%)	2.72	1.87–3.95
**Lipid-lowering Treatment**							
Mono therapy	808 (83.7%)	455 (84.4%)	110 (81.4%)	91 (75.8%)	1464 (83.2%)	0.80	0.60–0.92
Dual therapy	157 (16.2%)	84 (15.5%)	25 (18.5%)	29 (24.1%)	295 (16.7%)	1.08	0.82–1.43

## Data Availability

The data presented in this study is available upon request from the corresponding author.
